# Comparative Analysis of Fermented Flatbreads in the Horn of Africa and the Southern Arabian Peninsula: A Picture of Biocultural Diversity

**DOI:** 10.3390/foods15081333

**Published:** 2026-04-11

**Authors:** Erin Wolgamuth, Salwa Yusuf, Francesca Vurro, Antonella Pasqualone

**Affiliations:** 1 Research Group Transdisciplinary Agrifood Studies (TAFS), Department of History, Archaeology, Arts, Philosophy and Ethics, Vrije Universiteit Brussel, 1050 Brussels, Belgium; 2 Independent Researcher, Hargeisa JH09010, Somalia; salwacali08@gmail.com; 3 Department of Soil, Plant and Food Science (DISSPA), University of Bari ‘Aldo Moro’, Via Amendola 165/a, 70126 Bari, Italy; francesca.vurro97@gmail.com

**Keywords:** Yemen, Somalia, Somaliland, Ethiopia, Sudan, Saudi Arabia, *lahoh*, *kisra*, *injera*, hierarchical cluster analysis

## Abstract

Regular social, economic and agricultural interactions occurred between the Horn of Africa and Southern Arabian Peninsula for millennia, raising questions about potential geo-culinary exchanges, including of the little-studied fermented flatbreads produced in these areas. A comparative analysis of Somali *laxoox*/*canjeero*, Ethiopian *injera*, Sudanese *kisra* and Yemeni/Saudi *lahoh* was conducted by combining a literature review and consultations with 17 local experts, then processing the data in a hierarchical cluster analysis to quantify “biocultural” diversity. In an interdisciplinary approach, technical aspects (bread appearance, ingredients, and production stages) and cultural characteristics (consumption patterns and social function) were considered to identify key descriptors of the breads. A dendrogram generated through cluster analysis of a binary (0/1) matrix, structured with the key descriptors, showed that each bread has a distinct biocultural identity, and enabled the quantification of their similarities. Somali *laxoox*/*canjeero* and Yemeni/Saudi *lahoh* had a 64% similarity to each other (Jaccard index); each had a 53% similarity to Ethiopian *injera*; while all of them were 41% similar to Sudanese *kisra*. Hierarchical cluster analysis, applied for the first time to flatbreads, contributes to their comprehensive characterization and comparison in this unique geographic region and lays the foundations for policies to protect their identity and quality.

## 1. Introduction

Given that flatbreads are defined by a limited set of characteristics, their global diversity is significant [1]. The range of ingredients, tools, and processing techniques reflect the contexts, creativity, and experience of their cooks. Flatbread has had culinary and nutritional importance since the advent of agriculture and is likely among one of the first processed foods [2]. The Kitab al-Tabikh, a Middle Eastern cookbook written over one thousand years ago by Al Baghdadi [3], described six flatbreads and their distinct preparations [3].

While the variety of flatbreads from the Mediterranean region has been extensively investigated [4,5], fermented flatbreads from the Horn of Africa and the Southern Arabian Peninsula have been less studied, despite being a staple of the local diet and central to their respective cultures. There has been regular social, economic, and agricultural interactions between these geographical areas, which are less than 30 km apart at their nearest point along the Red Sea, since at least the Middle Stone Age [6,7,8,9,10,11,12,13,14,15]. Environmental similarities include latitude, the use of indigenous crops, and the successive adoption of foreign crop species [16]. Given this history, a basic assumption about historical foodway exchange is reasonable and raises questions about similarities among, for example, breads produced in these regions.

The diffusion of flatbreads from the Fertile Crescent followed the same path as cereals, spreading to the Mediterranean (including North Africa), the Arabian Peninsula, and beyond [17]. Today, flatbreads are the most common [18] among staple breads and significant to total nutrient intake in the Middle East and North Africa [1]. While wheat, as flatbread, is the modern-day foundation of the Middle Eastern diet [19,20], other grains, such as sorghum and maize, are important in the Arabian Peninsula where flatbreads are included in dishes which feature traditional ingredients such as mutton, dates and date syrup, sesame seeds, cardamom, or fennel [18]. Similarly, cereals of African origin, such as sorghum, millet, or teff, are used in breadmaking in the Horn. They do not contain gluten, so their flours are used to prepare bread batters typically fermented and baked on griddles [21]. Northeast Africa (Central Sudan and the Northern Ethio-Eritrean highlands, Sudan, Eritrea, Djibouti, Ethiopia, and Somalia) has served as a transition zone between African “porridge culture” and Near Eastern dough-based “bread culture” [22,23,24].

Per archeological evidence, the use of pottery in Africa began more than 2000 years before cereals were cultivated. In the Near East, evidence indicates the opposite—domesticated cereals were used 2000 years earlier than ceramics [24]. Pottery was essential to make and consume porridge, which continues as a dietary staple in much of Africa; in contrast, in the Near East, flat cakes were baked upon ash [24]. Rooted in the porridge tradition, a batter-based bread production of native grains emerged and continues in Northeast Africa [22]. Flatbreads produced from non-glutenous grains in the Horn of Africa is representative of this fusion of African and Near Eastern preparations. Sudanese *kisra* offers an interesting meeting point of these traditions: its multi-purpose batter is used to make both flatbread and *aseeda*, a popular porridge. While *aseeda* is also common in North Africa and the Middle East, this multi-purpose batter appears to be unique to the Sudanese context and neighbouring Chad [25].

The aim of this study was to analyze and compare in detail four batter-based, fermented flatbreads from the Horn of Africa and Arabian Peninsula: Somali *laxoox*/*canjeero*, Ethiopian *injera*/*taita*, Sudanese *kisra*, and Southern Arabian *lahoh*, which appears in Yemen and Saudi Arabia. The study defines the flatbreads individually, considering their quality characteristics, ingredients, production steps, consumption patterns, and cultural characteristics, and measures the degrees of similarity among them. Previous studies have compared limited aspects of *injera* and *kisra* [26], or *laxoox*/*canjeero* and *lahoh* [27], but no previous publication has extended a comprehensive comparison to the types of bread proposed here or considered cultural aspects. The results of this study will fill a knowledge gap of biocultural diversity among these artisanal fermented flatbreads, documenting cultural heritage and laying the foundations for food production and quality, culinary heritage and nutrition policies.

## 2. Materials and Methods

### 2.1. Approach

The fermented flatbreads analyzed were: *laxoox*/*canjeero* (Somaliland/Somalia), *injera*/*taita* (Ethiopia), *kisra* (Sudan), and Southern Arabian *lahoh* (Yemen and Saudi Arabia). The study included a detailed analysis of bread quality characteristics, ingredients, production steps (batter preparation, fermentation, shaping and baking), and consumption patterns, as well as cultural characteristics relating to the centrality of the investigated breads in local food systems and their social contexts. Data collection was based on review of secondary sources and fieldwork, the latter consisting of consultations with local experts. The study is limited to bread preparation from the last 50 years in the countries of focus with additional information on historical ingredients, tools, and techniques where available. In most cases, bread production has remained largely similar over the time period. Where differences were noted, they are mentioned with the corresponding location or era (e.g., the 1990s). As a tool for reference and comparison, relevant vocabulary is presented in Supplementary Table S1.

### 2.2. Databases and Sources

A comprehensive literature search was conducted via the Scopus database and print literature which described contemporary production of the investigated flatbreads from 1960 to 2025. In Scopus, the names of each bread with various spellings, i.e., *kisra* and *kissra* (Sudan); *lahoh*, *lahoh*, *lahuh*, *lohouh*, *lohoh*, *lohuh*, *laxoox*, *laxoox* (Somalia, Somaliland, Yemen, Saudi Arabia); *canjeero*, *anjero*, *anjeero* (Somalia); *injera*, *taita* (Ethiopia), as well as combinations of bread and country names with the words “fermented,” “fermentation,” “flatbread,” and “traditional flatbread” were searched in the title, abstract, and keywords. In some cases, additional searches were made, using specific ingredients as search terms, e.g., “teff,” “sorghum,” or local names such as “*jowar*” for sorghum. The results, in narrative form, were summarized for the purposes of comparing the breads under study.

### 2.3. Fieldwork

To clarify ambiguities or obtain details regarding information from published sources, 17 local experts (three from Saudi Arabia; three from Sudan; six from Yemen; one from Ethiopia; and four from Somaliland) were consulted between June 2024 and October 2025. They were selected via convenience sampling, starting with the authors’ in-country networks. The experts were selected primarily based on their status as longstanding home bread makers of the studied breads, and in some cases based on cultural proximity to the breads (i.e., being from the locations where the breads are regularly produced and consumed and therefore familiar with production and biocultural characteristics). Additional experts were scholars involved in the study of modern and traditional agri-food productions, including bread, in the region of focus. The majority were women over the age of 50, reflecting the key demographic with breadmaking experience across the region, although these were not selection criteria. All experts were informed about the purpose of the study and adherence to data confidentiality protocols. Their participation was voluntary and they had the option to leave the consultation at any time. Formal consent was obtained at the start of each consultation. Information was solicited from the experts via semi-structured, qualitative interviews. Given the political and conflict contexts of the studied region, most interviews took place remotely, lasting between 60 and 90 min. Notes were recorded by the researchers. In some cases, Supplementary Information (including oral input, video demonstrations and photographs) was provided by the experts through digital communications.

### 2.4. Hierarchical Cluster Analysis

Based on qualitative data from documentary sources and fieldwork, key descriptors were identified and used to create a 0/1 binary matrix—either present (1) or absent (0). These 69 descriptors (Supplementary Table S2) underwent hierarchical cluster analysis to generate a dendrogram of “biocultural” similarity. Hierarchical cluster analysis was performed using OriginPro software, version 2024 (OriginLab Corporation, Northampton, MA, USA). The Jaccard similarity coefficient was used to estimate the similarity among breads. The Unweighted Pair Group Method using Arithmetic Averages (UPGMA) procedure was used for analysis and to create the dendrogram.

## 3. Results

The results of fieldwork and literature analysis were examined to identify key descriptors of the four flatbread types in terms of appearance and quality characteristics, ingredients, production stages, consumption patterns, and social implications. The key descriptors are reported in Table 1.

### 3.1. Bread Appearance and Quality Characteristics

Key descriptors related to appearance and quality characteristics were: bread shape, size, thickness, colour, presence of patterns on the surface, and texture (Table 1).

**Figure 1:**
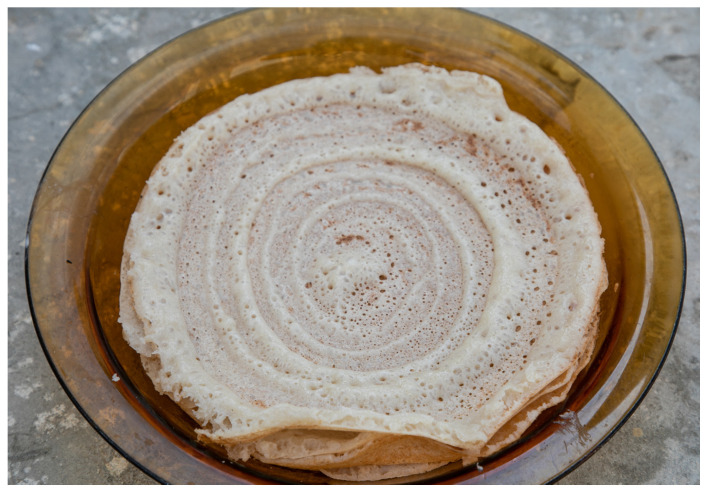
Somali *laxoox*/*canjeero* with its typical spiral pattern.

Somali *laxoox*/*canjeero* is spongy in texture and round, ranging in diameter from 13 to 20 cm [27,28]. It features a golden-brown colour on the underside touching the griddle, and a light beige to light brown colour on the upper side, which does not touch the griddle surface. Colour also depends on the unique formulation of the batch of bread [27,28]. The upper side features small bubbles, known as “eyes.” The upper side also typically features a distinct swirl pattern (Figure 1), created as the batter is spread across the griddle and which, due to the high viscosity of the batter, remains in the finished product. Contemporary *laxoox/canjeero* preparation encompasses varied production methods (e.g., the use or absence of a pre-gelatinized starter; a diversity of formulation) but all achieve a relatively uniform outcome in appearance.

**Figure 2:**
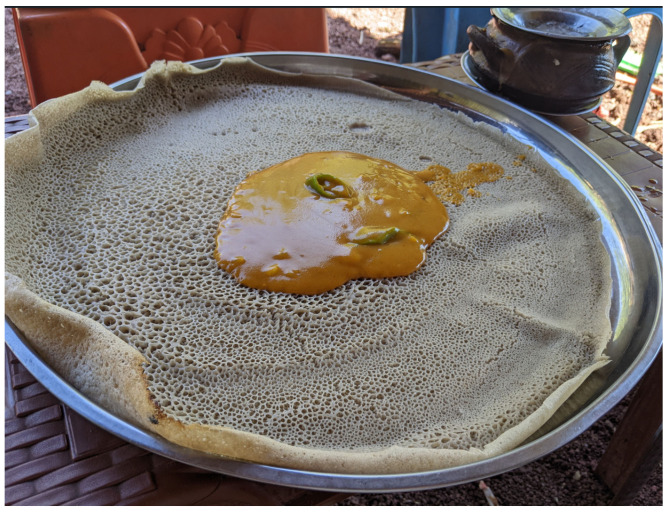
Ethiopian *injera* with *shiro*.

Ethiopian *injera* is a spongy, round bread of 50–60 cm in diameter. It features uniformly spaced “eyes” on the upper surface [29] (Figure 2). The Tigrayan bread known as *taita* is considered in the literature an equivalent to *injera* in quality characteristics, composition, production, and consumption, by a different name [30,31,32,33,34]. *Injera*/*taita* colour is uniform across the upper and undersides of the bread but varies from a rusty red to light brown or yellow depending on the variety of teff [35] and other ingredients used. Other quality characteristics include rollability and extensibility [36]. *Injera*/*taita* is between 2 [29] and 6 mm thick [37].

**Figure 3:**
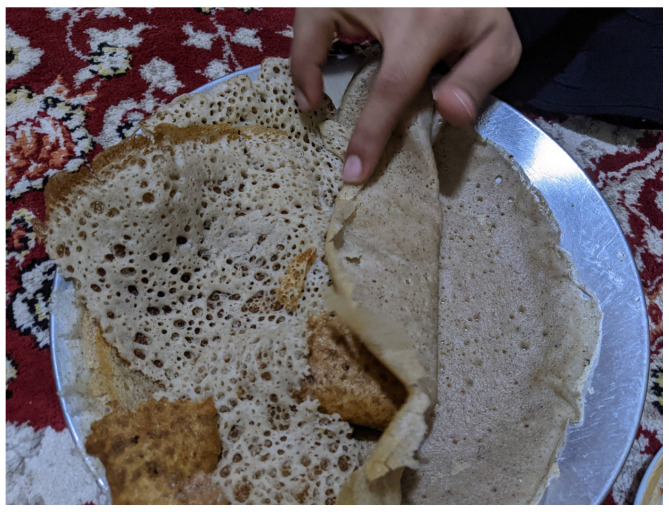
Southern Arabian *lahoh*.

The appearance of Southern Arabian *lahoh* is affected by a marked variation in preparation methods. A version from the northern regions and the Tihama (the hot, humid, and fertile Red Sea coastal plain, a vital agricultural region) of Yemen, also “famous” in Al-Mahwit governorate of the same country, is thick and moist. In contrast, Taiz (in southwestern Yemen) *lahoh* is “light as silk” and relatively dry [38]. Usually, *lahoh* has a circular shape and ranges in diameter from 15 to 30 cm, with an average thickness of 4 mm, although the size increases to 45 cm or larger when prepared for large groups on a larger griddle [27]. A recently studied sample of the elongated *lahoh Ariiqy*, originating in Aruuq Village near Taiz, was 55 cm in length and paper-thin [27]. The colour of Southern Arabian *lahoh* varies according to the ingredients used. In Saudi Arabia, it is typically off-white or yellow in colour, with a golden to dark brown underside. In Yemen the upper surface colour varies from similarly light to a deeper brown. *Lahoh* Ariiqy features a striped pattern with alternating off-white and dark red colours [27]. The bread commonly features many “eyes” on the upper surface which may be irregular in size and distribution (Figure 3).

**Figure 4:**
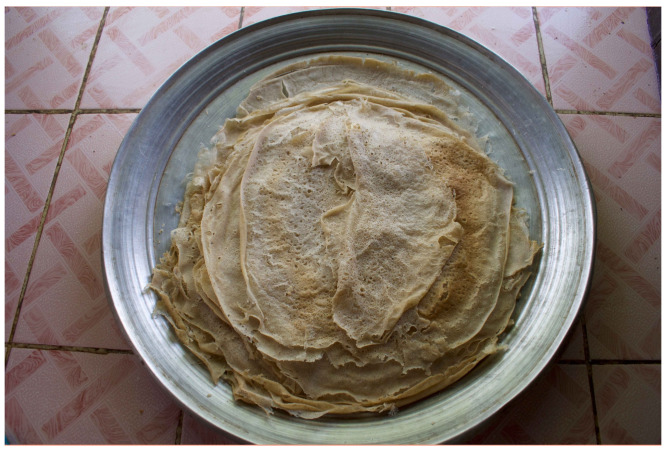
Sudanese *kisra*. Photo credit: Omer Al Tijani.

Sudanese *kisra* has changed little over the last few centuries [39], although its popularity is declining due to a preference for commercial and homemade breads that are easier to prepare [40]. This thin bread is 30–45 cm long and 1–1.5 mm thick [41] (Figure 4). Light brown to creamy white in colour, *kisra* is typically shaped on the griddle into a large oblong or oval form and then folded into a square shape for storage. Desirable quality features include an open-textured, regular cellular-type structure [41], but without the large, distinct “eyes” preferred in the other studied breads. Ideally, *kisra* is “supple, soft, and moist in texture, but not spongy” [41]. Different varieties include *kisra rhaheefa*/*rahifa* (very thin), *kisra-kerr* (thicker), and *kissra-habashiya* (“Ethiopian-style” *kisra*, more porous and spongier, with “eyes”) [42].

### 3.2. Bread Ingredients and Nutritional Composition

Key descriptors related to ingredients were the types of grains used and other, additional ingredients (Table 1). “Millet” is a common secondary ingredient among the studied breads. It is an umbrella term for various small-seeded annual grasses in the *Poaceae* family, comprising several taxa such as pearl millet (*Pennisetum glaucum*), finger millet (*Eleusine coracana*), proso millet (*Panicum miliaceum*), barnyard millet (*Echinochloa crusgalli*), little millet (*Panicum sumatrense*), kodo millet (*Paspalum scrobiculatum*), browntop millet (*Brachiaria ramosa*) and foxtail millet (*Setaria italica*). Pearl millet is the most widely cultivated in all sub-Saharan countries, while finger millet, which originated in East Africa [43], is typically cultivated in East, Southern, and Central Africa [44]. It would be of interest in future studies to discover the specific type of millet used for each type of bread.

**Figure 5:**
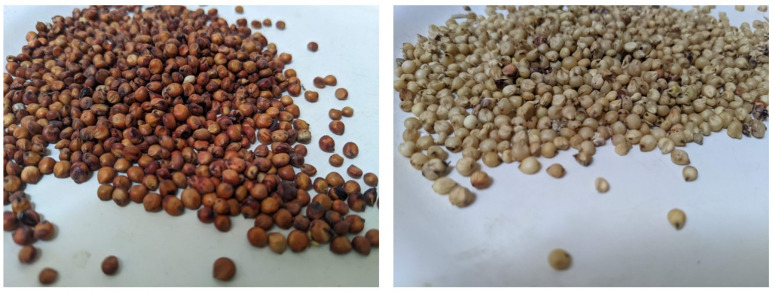
Red (Adan Gaab variety, **left**) and white (Cilmi Jama variety, **right**) sorghum from Gabiley, Somaliland.

Somali *laxoox*/*canjeero* formulation has evolved over time. In the early–mid-20th century it was prepared from sorghum (*Sorghum bicolor*) (Figure 5), which was hand-ground with water into a paste using a saddle quern stone (*mixdin*). Today, refined wheat flour (*Triticum aestivum* ssp. *aestivum*) is the most common ingredient [28]. In Somaliland and Ethiopia’s Somali State, soft wheat flour is typically included in combination with varieties of sorghum (in Somaliland, preferred local traditional landraces for *laxoox* preparation are *masago*, *carabi*, and *abaduri*) [45], durum wheat (*Triticum turgidum* ssp. *durum*), barley (*Hordeum vulgare*), maize (*Zea mays*), millet (mainly pearl (*Pennisetum glaucum*) and finger (*Eleusine coracana*)), cowpea (*Vigna unguiculata*), groundnut (*Arachis hypogaea*), and teff (*Eragrostis tef*) [27]. A breadmaker’s unique combination of dry ingredients is known as *budo*. Geographic differences in ingredients are observed as well. Some households, primarily in South–Central Somalia, use only (mainly refined) wheat flour or a mix of wheat and maize flours. Other ingredients are relatively standard across geographies and include salt and water, with occasional flavourings such as fenugreek or black cumin (Nigella seed) [27], coriander, or sugar [28].

Ethiopian *injera*/*taita* is known for its primary ingredient, teff. There is a common perception that teff *injera* is an ancient food, based largely on the coincidence of archaeobotanical evidence of teff cultivation and archeological evidence of a clay tray similar to the *injera* baking griddle, both of which date to circa the sixth century CE [34,46]. Yet, it is not confirmed that teff *injera* was commonly consumed in that era. The earliest evidence of teff *injera* as a staple food comes from Northern Ethiopia in the early 1750s; the bread was not even introduced to other local ethnic groups until the 19th century CE [33]. In the last 60 years, the use of other cereals such as millet, barley, wheat, sorghum, or maize has been documented [47]. Barley is commonly used to prepare *injera* in the highlands, and sorghum [48], maize or millet [49] in the lowlands. Taken together, this data indicates that *injera*/*taita* is more diverse in composition than is popularly acknowledged.

Nevertheless, teff persists as the iconic grain of *injera*/*taita*. White teff symbolizes wealth as opposed to the cheaper, more common red teff [34], a preference recorded in a description of *taita* in an 1844 journal entry at Adwa, in present-day Tigray Region, where peas and vetches were also incorporated [50]. Another potential basis of consumer preference for white teff comes from a recent study on the optimization of *injera* quality characteristics [36]. The study found that red teff had a negative impact on “eye” distribution, extensibility, and texture compared to non-red teff varieties. Furthermore, a consumer trend has arisen of adding a few grams of rice flour to the *injera* batter to lighten the colour [29]. Stone milling is the traditional present-day cottage grain milling system [51]; in the mid-19th century, a saddle quern stone raised upon a clay–pebble foundation (*mouthan*) was observed for the same purpose [50]. Other ingredients include salt or flavourings such as fenugreek [47].

Southern Arabian *lahoh* features red and white varieties of sorghum and pearl millet, which were characterized as the “most important” *lahoh* ingredients in an interview with a local expert. In ancient Yemen, sorghum and millet were typically used to produce pancake-like breads in a context of limited fuel, hot climates, and low-gluten wheat [52]. Sorghum may be a longstanding essential ingredient in this bread, as evidenced by the 10th century BCE writings of al-Hamdani [53], who remarked “a venerable ancestor said that [Yemeni] *lahuh* is sorghum bread cooked on a griddle…” [54]. Sorghum and millet (pearl and finger millet) were commonly cultivated in Southern Arabia [54]. Adra [55] reported that the major crops grown in the Yemeni highlands were sorghum (*dhura*), maize (*rumi*), wheat (*burr*) and barley (*sha’ir*). Today, maize, barley, and wheat are among Saudi Arabia’s primary crops [56] and carry into breadmaking.

Other ingredients, supported by the literature and mentioned in expert consultations, included refined and whole wheat, maize, millet, and/or barley [54]. One traditional Saudi version included only pearl millet and water in a 1:2 ratio with a microbial starter [57], while modern versions may include salt and leavening agents such as baking soda and yeast, per experts. Bornstein-Johannsen [58] noted that yellow (*safre*) and white sorghum (*baithe*) were preferred for baking bread in Yemen. At the time of writing (1970s), Bornstein-Johannsen further observed that while sorghum and millet flours were often mixed, they were never combined with barley or wheat; today, these cereals are readily blended in *lahoh*. In a traditional Yemeni preparation (one to two generations previous in urban areas and continuing today in some rural areas), household cooks soaked whole grains in water and hand-milled them using a rotary or saddle quern stone known as a *madhana*. Today, dry ingredients may be available pre-milled or using fee-based small-scale commercial mills. Water and salt are added to the batter. Other ingredients used by Yemeni sources may include fenugreek or *za’atar* ([59]; interviews). *Lahoh* from Taiz (Yemen) may include skimmed cow’s milk while *lahoh mubayyid* includes eggs [38].

Any variety of sorghum may be used in Sudanese *kisra*, but white varieties are preferred, whereas red or brown varieties, such as *Trica*, are preferred for porridge ([60]; expert interviews). Popular options are locally known as *feterita* (white grain colour), *tabat*, *dabar* (tan colour), *orbit* (tan colour), *safra*, or *ahmer*, to name a few. Sorghum grains can be mixed with 1 to 15% *w*/*w* of wheat or millet grains and milled together; alternately, these quantities of pre-milled flour are combined ([60]; expert consultation). In Western Sudan (Kordofan and Darfur), *kisra* is prepared with millet instead of sorghum [61]. In some areas, sorghum has been fortified during at least the last half-century with wheat or millet flour to improve bread quality [60]. While *kisra* provides most of local dietary proteins and energy, especially in rural areas of Sudan [62], recent studies propose innovative fortifications, such as the addition of locally grown bambara groundnut [63]. In communities with less access to mechanized tools or pre-milled ingredients, dry hand milling is performed with a wooden mortar and pestle, while stone mills are used in urban areas [61]. Water is typically the only additional ingredient, although one expert shared that she sets a white, transparent towel over the *kisra* batter ready for fermentation, and scatters black cumin and fenugreek seeds on top of the towel. These seeds, rich in phytochemicals, impart flavour and prevent spoiling.

Although formulation varied across the breads, their nutritional composition substantially overlapped (Table 2). A slightly wider range of variation was observed for fibre in *injera*/*taita*, and slightly lower values for the same parameter were found in *kisra*. However, nutritional profiles warrant further investigation; because these data were aggregated from a diverse literature, comparability may be limited by inter-laboratory variations in analytical conditions.

### 3.3. Production Stages

**Figure 6:**
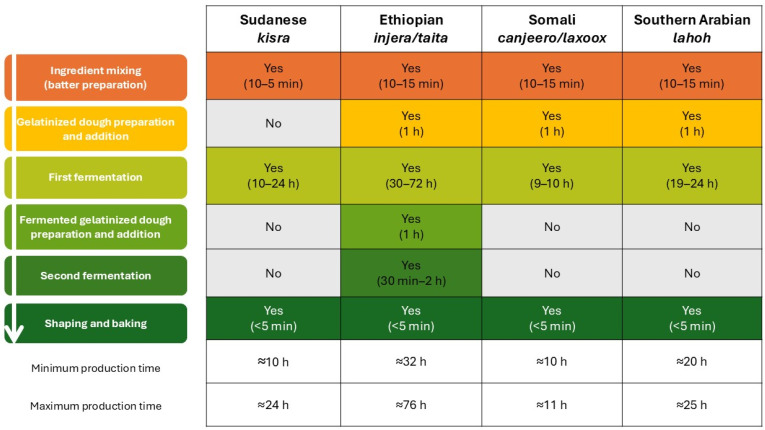
Production flowchart and stages observed in four flatbreads: Ethiopian *injera*/*taita*, Sudanese *kisra*, Somali *canjeero*/*laxoox*, and Southern Arabian *lahoh*. Duration, varying among breads, is specified in each box.

The main stages of production are summarized in Figure 6, where the flowchart is combined with a table showing which stages are carried out for each type of bread, and the typical duration per stage and per bread.

### 3.4. Batter Preparation

Key descriptors related to batter preparation were the density of the batter and the possibility of dual use (Table 1).

In the preparation of *laxoox*/*canjeero*, Somali household cooks combine all dry ingredients (except for salt, which is added at the time of baking) in a container, usually plastic and used also for leavening. The flour mixture is hydrated with room temperature water at a ratio of 1:2 *w*/*v*. Warmer water may be used to activate fermentation in cold weather. Sometimes, a microbial starter known as *dhanaanis* is added to catalyze fermentation. *Dhanaanis* consists of a portion of the previous day’s batter and/or the use of the unwashed fermentation container from the previous batch of bread which retains a film of batter on the inside. *Dhanaanis* is commonly used in Somaliland and Ethiopia’s Somali State. In South–Central Somalia, *cajiin*, a pre-gelatinized hot water dough, may be added to the batter. *Cajiin* was prepared by hand from sorghum flour in the early–mid-20th century across greater Somalia (including Somaliland). Today it is used only in Southern, Central, and Eastern Somalia and is prepared with commercial-grade sheeting machines and sold in retail portions in marketplaces [28]. *Cajiin* would have played an important role in earlier *laxoox*/*canjeero* preparation by enabling a soft, dough-like texture from low-gluten or non-glutenous grains such as sorghum. Today its use often coincides with refined wheat (i.e., gluten-containing) flour and is therefore technically unnecessary to achieve the desired texture. The batter is thoroughly mixed by hand to form a fluid but viscous mixture. Today, in some urban locations, batter may be mixed using a household blender.

To prepare Ethiopian *injera*/*taita* batter, dry ingredients and water are combined in a 1:2 ratio [47,49] along with *irsho* (also spelled *ersho*), a microbial starter saved from a previous fermentation between two and seven days prior [47]. The ingredients are mixed thoroughly by hand to form a thin paste in a fermentation container (*bohaka*) made of clay, metal, or wood [66]. A pre-gelatinized dough (*absit*) is prepared and added to the batter after the first of two stages of fermentation (Section 3.5).

For Southern Arabian *lahoh*, a gelatinized dough (*shorba*) is traditionally prepared (especially in rural areas) by mixing flour with hot water in a 2:3 *w*/*v* ratio. Then, the *shorba* is added to the rest of the dry ingredients and the resulting mixture is combined with warm water at a ratio of 2:3 *w*/*v* to make the batter, known as *ajiin*/*ajeen*. Additional water may be added, if needed, to achieve the thin consistency required to prepare the flatbread. Today, *lahoh* batter may not include *shorba*, given the modern-day use of glutinous cereals, like wheat, to achieve the preferred texture. Saudi *lahoh* batter is prepared by mixing the dry ingredients and water in a 1:2 *w*/*v* or 1:3 *w*/*v* ratio with a 5% microbial starter to yield a smooth, thin mixture, usually without *shorba*. Modern versions may include instant yeast, baking powder, or salt. Mixing is done by hand (traditional/rural), with a whisk, or in a blender (modern).

Sudanese *kisra* is prepared by mixing flour with room temperature water in a ratio of between 1:2 *w*/*v* and 2:3 *w*/*v* [39,42,60]. *Kisra* batter incorporates a microbial starter from an established “vigorously fermenting dough” [60], added to a degree of between 5% [67] and 10% [39]. One expert reported the use of yoghurt (around 5%) to facilitate fermentation when a microbial starter is not readily available, an observation further substantiated by the literature [68]. The resulting, thick batter is immediately used to prepare porridge or is thinned with water at a later step (after fermentation) to produce a pourable batter, known by the Arabic *ajiin* (العجين), for breadmaking [39,62]. *Kisra* ingredients are combined either in a plastic bucket or bowl (*jardal*) or in the more traditional *kantuush al ajiin* carved from wood or *garae* carved from squash, per experts. The ingredients are mixed using a long, wooden tool with a t-shape at the top, called a *mufraka*. Large quantities of *kisra* are prepared for gatherings such as weddings; in these instances, six to seven women may assemble to prepare the batter in a 50 L steel pot known as a *halla* (expert consultation).

### 3.5. Fermentation

Key descriptors related to fermentation were the number of fermentations carried out and their duration, the use of a microbial starter and of pre-gelatinized fermented dough (Table 1).

Somali *laxoox*/*canjeero* typically relies on a single, long (average 9.5 h, typically overnight), spontaneous fermentation by wild yeasts. In Somaliland, fermentation is enhanced by a microbial starter known as *dhanaanis* (described in Section 3.4); in South–Central Somalia, *dhanaanis* is used only as needed, e.g., in colder seasons, due to a preference for a less sour taste [28]. To adapt to changing seasons and weather, household cooks change the fermentation location (e.g., warmer locations such as the kitchen) or wrap the fermentation container in cloth to retain heat. If, near the time of baking, the batter is still under-fermented, cooks add hot water or tea, additional *dhanaanis*, or a portion of fermented batter from a neighbour. If the batter is perceived to be over-fermented, based on a strong sour smell, then cool water is added.

The fermentation of Ethiopian *injera*/*taita* is a two-stage process. As in other indigenous Ethiopian fermented foods and beverages, it is an acid–alcohol fermentation [49]. The first stage takes 30–72 h depending on the concentration of *irsho*, the altitude, and the *bohaka* material [35,49,66]. To encourage fermentation, the *bohaka* is not washed thoroughly; a film of fermented batter is left behind [49,66]. The fermented batter separates into sediment and *irsho*, a supernatant yellow liquid that is poured off and saved as a microbial starter for the subsequent batch of *injera*/*taita*. Gas production is also considered a sign of successful fermentation [66]. A variety of *injera* known as *aflegna* is fermented in the first stage for only 12–24 h. Popular in rural areas but also prepared in urban locations when the need for *injera* is urgent, *aflegna* has a sweeter flavour and aroma, and a red underside [66]. Although the fermentation time is considerably shorter than typical *injera*, *aflegna* ferments much longer than the compared breads. A third type of *injera*, known as *komtata injera*, is made from over-fermented batter.

Following the first fermentation stage, a portion of the batter is separated, mixed with water at a 1:3 *v*/*v* ratio [66], and boiled for 2–5 min [49]. It is then cooled to around 40 °C and returned to the batter in the *bohaka* [47]. This pre-gelatinized dough, called *absit*, binds and helps retain gas in the dough; without it, the *injera* lacks its characteristic “eyes” [66]. *Absit* also confers the preferred soft texture on gluten-free, teff-based *injera* (among the common alternative cereals, millet and corn also require *absit*) [66]. After the addition of the *absit*, the batter is fermented again in the *bohaka* for 30–120 min [35]. The twice-fermented batter is thinned with water if needed [35]. In a detailed, mid-19th century description of *taita* preparation, Parkyns [50] describes a single long fermentation in an earthen jar “for a day and a night to leaven: it is then ready for baking.”

Among the “countless” recipes for Southern Arabian *lahoh*, the main differences usually pertain to the quantity of yeast and fermentation time [69]. Yemeni breads typically include a microbial starter (*dhanaanis*) [58] if *lahoh* is prepared on a regular basis, for example every night of the month of Ramadan; otherwise, the infrequent preparation of this bread makes *dhanaanis* unnecessary to maintain. One home cook, preparing a starter from scratch, fermented it for six days [70]. In traditional versions of *lahoh*, either the batter (*ajiin*) and pre-gelatinized dough (*shorba*) were long fermented separately overnight (10–12 h) and combined in the morning, or the *shorba* was heated and added while hot to the *cajiin* batter to ferment overnight. In Saudi Arabia, the batter with microbial starter may be fermented in a warm place for as long as 18–24 h for a sourer taste [57], or a tangy flavour [71] “as sour as vinegar,” as noted by one expert. In modern preparations which include instant yeast, the batter ferments for 10–20 min. The appearance of a clear liquid layer (in both modern and traditional preparations) is an indicator of successful fermentation and the batter’s readiness to cook; the liquid is mixed into the batter.

Once a microbial starter is added to Sudanese *kisra* dough, fermentation proceeds for 12–24 h depending on the temperature and the tannin content of the sorghum cultivar used [39,41,42,57,60,62,72,73]. Fermentation occurs in a round, clay vessel (*khomara*) of a size suitable to feed the family [60] or a plastic container (*jardal*). In the past (previous two to three generations), a thin, wooden bowl known as *kantuush al ajiin* or *al qare* was used to mix and ferment the dough, according to experts. Fermentation may be accelerated (12–20 h at ambient temperature) with a small portion of microbial starter (*khmar*) or *roob*, a homemade curdled milk [62]. An expert explained that, if a microbial starter is not available, fermentation takes two days. When the preferred, fine texture develops in the later stages of this long fermentation [60], the dough is thinned with water to create a batter called *ajin* for baking [42,62]. If a layer of clear liquid appears on the batter surface during fermentation, it may be poured off or mixed in depending on the preference of the cook, per local experts. Bubbles on the batter surface indicate that it is ready to bake. One expert shared that in some areas of Sudan (including Khartoum) within the last twenty years, a pre-fermented, frozen *kisra* batter has become available for retail purchase; additional flour and water are added at home, and the batter is immediately ready to bake.

### 3.6. Shaping and Baking

Key descriptors related to shaping and baking were the way the batter is poured on the baking surface and the type of baking system used (Table 1).

**Figure 7:**
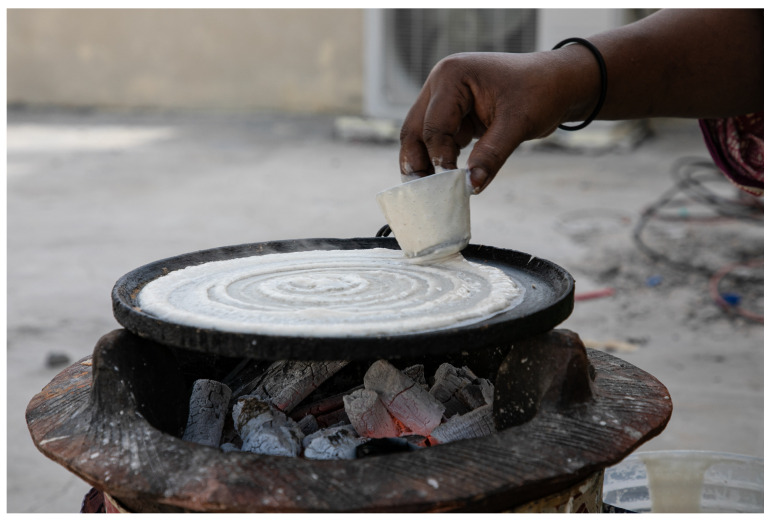
Shaping the batter of Somali *laxoox*/*canjeero* on the baking surface with the help of a plastic cup, following a spiral motion. Photo credit: Abdikarim Omar.

To shape Somali *laxoox*/*canjeero*, the cook scoops batter from the fermentation container using a flat-bottomed plastic cup and pours it onto the centre of the griddle. With the bottom of the cup touching the batter but not pressing through it, she quickly pushes the batter from the centre to the edges of the pan in a spiral motion (Figure 7). This technique produces a spiral effect in the upper surface of the bread which is retained in the finished product.

The baking bread is covered with a domed aluminum lid and steam-leavened, creating an open, spongy structure with “eyes.” When the underside of the bread is golden-brown it is removed from the griddle. Cooking time ranges between 2 and 5 min, though household cooks use different cues (e.g., the sound of condensation dripping from the lid onto the hot pan, or the smell of cooked batter) to assess doneness.

**Figure 8:**
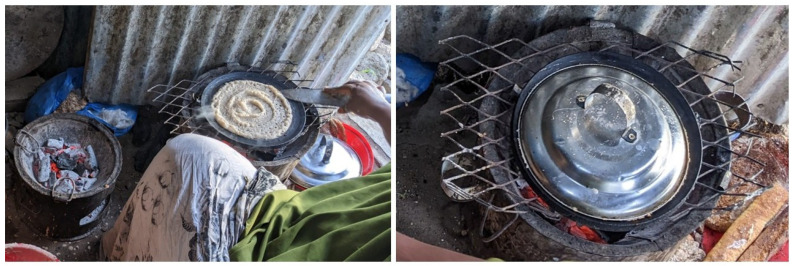
Somali *laxoox*/*canjeero* baked on the traditional cast iron griddle (*dhaawe*) atop a charcoal firebox (*girgire*) (**left**). A lid is used during the baking process (**right**).

Somali *laxoox*/*canjeero* is typically baked atop gas stoves or charcoal fireboxes (*girgire* in Somaliland/Northern Somalia and *burjiko* in Southern Somalia) (Figure 8), although the global production style may feature different materials and heat sources. The cast iron griddle (*dhaawe* in Somaliland and *bir canjeero* in South–Central Somalia) is flat, approximately 16–20 cm in size, and has short sides and a handle. The griddle is heated over medium–high or high heat and prepared with vegetable oil or ghee using a black cloth known as a *masaxaad* or a folded piece of flatbread, usually the first cooked piece. This reduces sticking and may be reapplied as needed. Some cooks rub large flakes of salt on the pan to further reduce sticking.

Ethiopian *injera* batter is poured onto the griddle in a circular motion using a plastic jug (*jog*) from the outer circumference inward to the centre in a reverse-spiral motion. The present-day technique for pouring batter onto the *mitad* was preceded (c. mid-19th century) by a hand-spreading technique, and the timing of the emergence of the spiral technique is not known [33]. A corresponding mid-19th century tool in Tigray Region is a jar made from a small calabash (*bourma*) [50]. The thin batter spreads across the pan to form a flat layer. Bubbles form on the surface within seconds, and the griddle is covered with a tightly fitting lid (*kidan*) and baked for 2–3 min [47] until the desired colour is achieved and “eyes” form. Only the underside is in contact with the griddle; the upper side of the bread is steam-leavened.

A large cast iron griddle (approximately 60 cm in diameter) is used to bake *injera*, called a *mitad* (Amharic) or *magogo* (Tigrinya). To reduce sticking, it may be prepared with crushed oily seeds [24,50], e.g., kale or cotton seeds [49], or lightly oiled [47]. The *mitad*/*magogo* is maintained at a relatively high heat (90–95 °C at centre) [47,49]. In Ethiopia, 96% of the population uses traditional biomass for cooking [74], and 50% of that energy is dedicated to baking *injera* [29]). In Northern Ethiopia (Tigray National Regional State and Wollo, part of Amhara National Regional State), traditional, enclosed baking stoves are used to bake *taita* [29]; the stoves are built permanently into the ground or on a raised platform of stones and mud and typically include two smoke outlets. Once cooked, the *injera* is lifted from the griddle by hand and cooled on a straw plate. *Injera* is stored for 2–4 days in a cylindrical, woven grass *mesob* with a flat bottom [47,49].

Per the traditional method of baking Southern Arabian *lahoh*, still in use today by some, the cook scoops and pours batter from a stout metal cup with a hole drilled in the bottom. Pouring in a spiral pattern, the cook starts from the centre of the griddle and spirals outwards or vice versa. The respondent who demonstrated this traditional method added that one generation earlier, the cup was pressed lightly into the batter and used to spread the batter across the griddle to create a spiral pattern (similar to Somali *laxoox*/*canjeero*). In the late 1970s in a Sana’a marketplace, a cook used a small gourd cup with a hole in the bottom to pour batter onto the griddle, per one expert. The bread is baked on one side for approximately one minute and the griddle is not covered. Only the underside of the flatbread touches the griddle and is baked until golden brown, although some household cooks turn the bread over to achieve an attractive colour on both sides.

**Figure 9:**
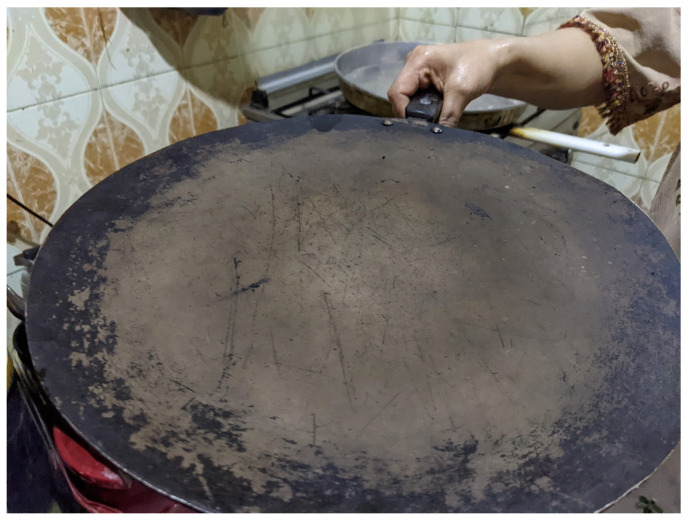
Metal disc (*dawa* or *sulla*) traditionally used in Yemen for baking *lahoh*.

While some flatbreads in Southern Arabia are prepared in a vertical *tannur* oven, *lahoh* is baked on a metal pan or disc called a *dawa* or *sulla* in Yemen [21] (Figure 9) placed on top of the *tannur* [58] or over a fire in an underground stove in Saudi Arabia, per one expert (for a detailed description of traditional baking systems, see Pasqualone [17]).

According to consulted experts, traditional *lahoh* for large groups is prepared in Yemen on a large metal *dawa* between 75 cm and 1 m in diameter. Modern Southern Arabian *lahoh* may be prepared over a gas stove using a *dawa* or another pan type, including nonstick. A high level of heat warms the pan quickly and is then reduced to medium. Ghee (*samn*) or vegetable, sesame, or olive oil may be applied to the pan before cooking and between breads. In Yemen it is applied with an oiled cloth called a *masah*; this was also observed in 1978 al-Ahjur, northwest of Sana’a, per an expert.

The process of baking Sudanese *kisra* is known as *awassa.* A very thin layer of batter is poured onto the griddle in a circular [41] or back-and-forth motion of wide, overlapping strips (expert consultation). A tool made from a date palm leaf or plastic (*ghegeriba*) [75], or a strip of wood [73] is used to spread the batter quickly and evenly across the surface into a thin sheet [42,60,72,73,76]. To prevent sticking, a thin layer of sesame oil or spinal fat (*taouk*) ([39,60], and expert consultations) is spread on the baking plate. After baking uncovered for 30 s to 2 min, and once the edges of the bread lift slightly from the griddle, the *kisra* sheet is removed; only one side of this thin bread touches the surface. The griddle plate is scraped after each piece of *kisra* is baked, to clear crumbs [75].

**Figure 10:**
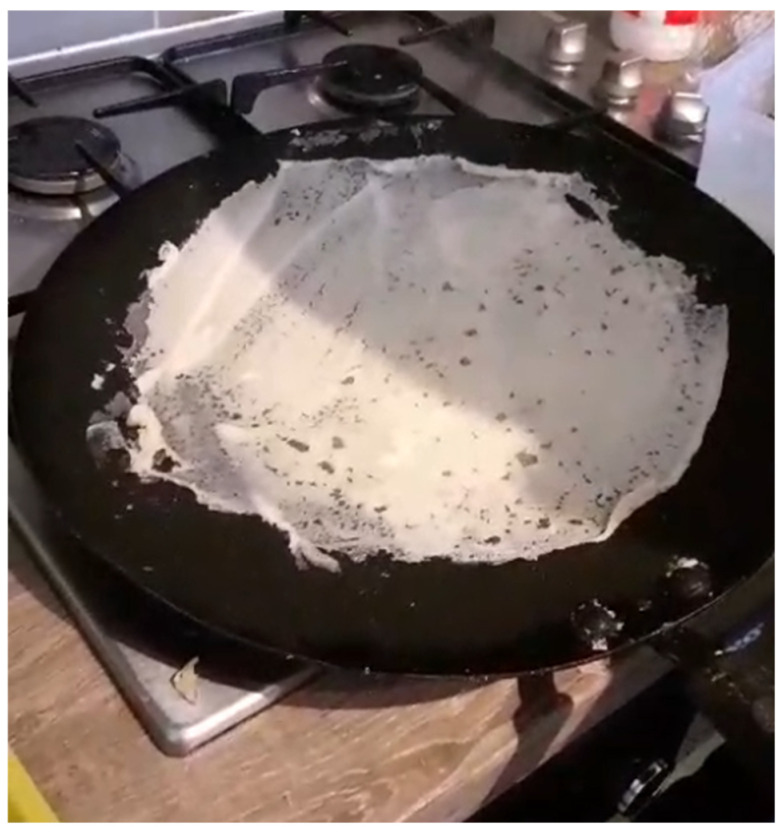
Sudanese *kisra* baked on a metal plate (*doka* or *saj awassa*, or *saj*) on a gas stove.

**Figure 11:**
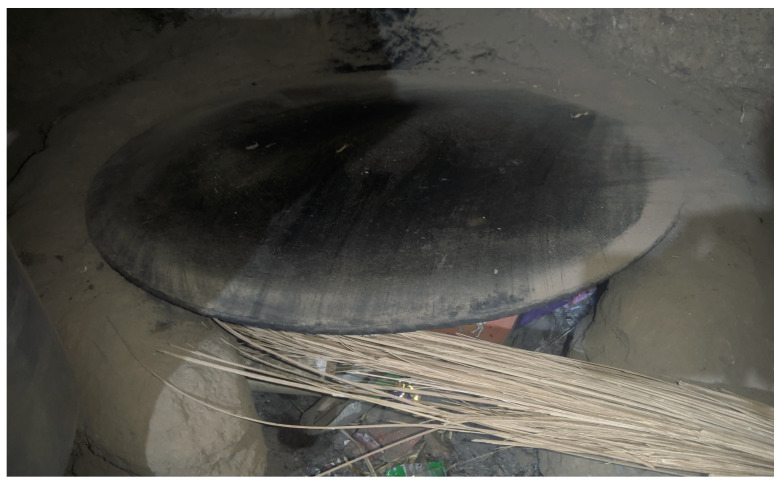
Traditional Sudanese griddle over a wood-fired clay stove. Photo credit: Mohamed Ahmed Dawoud.

To bake this bread, a metal or steel plate without raised edges (*doka* or *saj awassa*, also referred to simply as *saj*) is heated atop an electric or natural gas stove [42] (Figure 10) or over a wood-fired clay stove (Figure 11). *Saj* griddles may come in circle, square, or triangle shapes but are always large compared to other griddles as large sheets of *kisra* are expected to feed families and visitors, according to experts. Traditionally, a clay plate was used atop coal or wood [42] while rare granite *doka* have been reported [26].

### 3.7. Bread Consumption Patterns and Social Function

Several key descriptors were identified for consumption patterns regarding mealtimes, the way breads are served, accompanying dishes and social function (Table 1).

**Figure 12:**
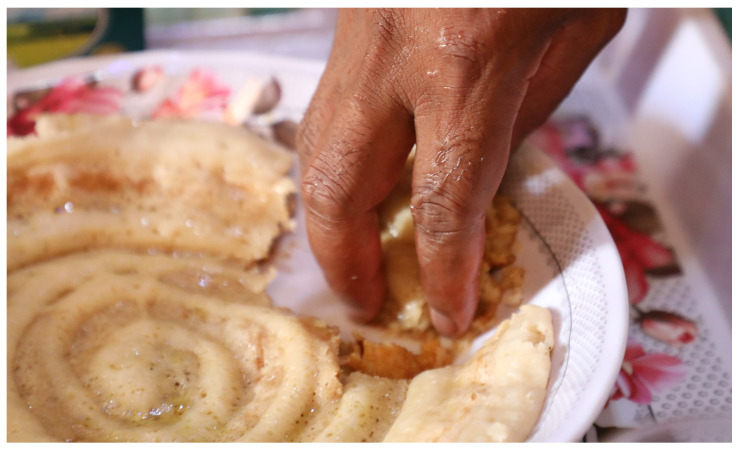
Traditional way of using Somali *laxoox*/*canjeero* as a utensil to pick up and soak up food and sauces.

Somali *laxoox*/*canjeero* is most often served at breakfast, usually with sweet Somali tea and sometimes drizzled with vegetable or olive oil, honey, or sugar [28]. Some pour tea over the *laxoox*/*canjeero* and eat it saturated in the hot liquid. For breakfast it may also be served with sauteed meat or eggs. The flatbread is sometimes eaten at lunch or dinner with various meat stews, vegetables or legumes. Pieces of *laxoox*/*canjeero* are torn off the main flatbread and used to pick up food and soak up sauces (Figure 12). *Laxoox*/*canjeero* is a popular food to break the day’s fast during the Muslim holy month of Ramadan. Apart from the habit of pouring tea onto *laxoox*/*canjeero*, it is otherwise served alongside lunch and dinner dishes but not underneath them, as is the case for Ethiopian *injera*/*taita*.

Ethiopian *injera*/*taita* is served at all mealtimes [21]. It is a daily staple for Tigray farmers who consume large quantities per meal [31]. *Injera* is served both under prepared food as an edible, communal platter, or alongside it in rolled pieces. *Taita* is served in layers on communal trays or baskets with vegetable or meat sauces ladled on top [34]. This bread is eaten with one or more types of sauces (*wot*) which may be spicy or mild, a lentil stew (*shiro*), or red pepper and vegetable oil (*silsi*) sometimes prepared with eggs, potatoes, or tomatoes [31]. *Wot* are legume-, vegetable- or meat-based and may contain a significant amount of vegetable oil or more expensive traditional butter (*qibe*). The texture of *injera* enables its use as a utensil. Approximately one-third of Ethiopia’s population is Muslim, and *injera* features at the *iftar* meal during Ramadan [77]. It is also a central element of Orthodox religious celebrations such as Christmas and Easter [47].

Al-Zabidi [78] remarked in the 13th century that *lahoh* was eaten in Southern Arabia with milk and sometimes with broth. Today, *lahoh* is consumed with a variety of dishes at different meals. One expert from Jizan, Saudi Arabia, emphasized the suitability of *lahoh* for communal meals. Prominent in traditional breakfasts ([59,61], and expert consultation), *lahoh* is covered with butter, ghee, or honey or eaten with a sweet, clotted cream (*qishta*), honey or cheese [71]. *Lahoh* is eaten at lunch or dinner with soup or stew, such as *molokhia*, made with jute leaf (Jew’s mallow, *Corchorus olitorius*) and eaten widely in the Middle East and North Africa, or *bamia*, a spiced tomato stew made with okra and lamb. It is also a key element of *shafut*, a dish of *lahoh* soaked in *laban* (soured milk) with herbs and fresh vegetables such as green pepper, onion, tomato, or cucumber ([58]; expert consultation). *Lahoh* may be served alongside *hilba*, a fenugreek-based condiment, or as a snack with *basbas*, a dry spice mixture, according to local experts. Southern Arabian *lahoh* is also a typical Ramadan bread. In addition to home preparation, *lahoh* is sold in large chain grocery stores, as reported by experts, and as street food [79].

Each piece of Sudanese *kisra* is traditionally folded into a rectangular shape and stored on a covered tray (*raika*) made from date palm leaves [60]; today it is preferably stored in an airtight container to keep for several days [39]. Many sheets of *kisra* are typically folded, stacked, and served under or alongside meat and vegetable stews (known as *edam*) [62], such as *Umm Bullot*, *Umm Sha’ifa*, and *Sukhina* edams, and sometimes *Al-Sharmout*, which means dried meat [61], or *molokhia* or *bamia*, as in the case of Southern Arabian *lahoh*. *Kisra* in Central Sudan may also be taken with fresh okra and mallow cooked with meat (fresh or dried), or with a soup made from dried, ground okra and meat [80].

### 3.8. Production Capacity and Social Dimension

The key descriptors relating to production capacity and social dimension were the typically small-scale artisanal nature of production and the central role of women in breadmaking (Table 1).

All the breads under study are prepared in the home or for cottage industry by women and/or girls ([34,39,41,62,72,81]; and expert consultations). In Sudan, for example, many household cooks have entered small-scale commercial production of *kisra* as technology and urbanization have advanced [82]. The (urban) informal economy emerges from economic need and marginalization; in Africa, most actors in the informal economy are women [83] whose production styles and ingredients resemble preparation for families and serve as a relatively consistent basis for analysis. These flatbreads may also be prepared—including by men—in full-scale restaurants or larger commercial settings; however, significant differences in ingredients and tool use and other aspects of production put those breads beyond the scope of this study.

In Sudan, so close is the association between women and *kisra* that the name for a woman or girl who prepares the bread, *awassa*, is the same word for the process of baking *kisra* [39]. Some of the articles referenced in this study reflect traditional preparations by housewives, relying on female experts to produce reliable bread samples for analysis in a research laboratory, including for *kisra* [41], Saudi *lahoh* [57] and Somali *laxoox* [27]. In Yemen, “*women have unique skills and experience in preparing lahoh…Girls may learn from a young age how to handle ingredients and prepare them with traditional skill …and they may introduce new tools…*” [59]. In some cases, this has evolved into women-run home-based artisanal production for sale or cottage industry [60,79,82]. In a late 1970s Sana’a marketplace, women sold *lahoh* for one riyal each, per one expert. In the Yemeni highlands, *lahoh* is a Ramadan fixture largely because it does not require the hard work of baking in a *tannour* oven and is therefore easier to produce by women who are fasting, per experts.

Women have vital roles elsewhere in the breadmaking value chain as well. In Somalia and Somaliland, women participate in harvesting some crops twice per year, and in processing cereals in preparation for breadmaking and other foods. This includes threshing, cleaning, winnowing, drying, and crushing or milling grains with quern stones or wooden mortars and pestles. Women are prominent vendors in retail grain markets in major cities and smaller towns, villages, and trade points, while men operate the small-scale mechanized mills which serve grain market customers. In Yemen, women are involved in a range of steps in grain cultivation, often in cooperation with men where respective duties are clearly delineated; nevertheless, women are uniquely responsible for cleaning grains, processing flour and preparing food [55]. In Sudan, per local experts, these mills (*ajana*) are rarely run by women. Men also operate mills in larger cities in Saudi Arabia.

### 3.9. Data Elaboration

**Figure 13:**
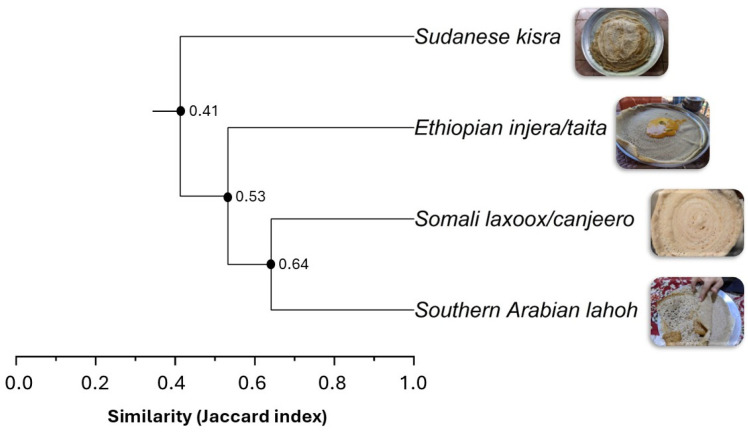
Dendrogram illustrating the degree of “biocultural” similarity among the four bread types under study.

The key descriptors summarized in Table 1 and illustrated in Section 3.1, Section 3.2, Section 3.3, Section 3.4, Section 3.5, Section 3.6, Section 3.7 and Section 3.8 were used to establish a binary (0/1) matrix with 69 entries (Supplementary Table S2). The entries underwent a hierarchical cluster analysis which was used to generate a dendrogram of biocultural similarity (Figure 13).

To better understand the effects of the different groups of descriptors (listed in Supplementary Table S2) on overall similarity, individual dendrograms were generated based on the following technical parameters, selected to maintain a similar number of entries (between 16 and 19): physical and nutritional parameters (bread appearance, texture, and nutritional composition; total = 19 entries), process-related parameters (batter preparation, fermentation, shaping and baking = 16 entries), and ingredients (primary and secondary; total = 17 entries). Compared to Figure 13, the structures of the individual dendrograms did not change; that is, the cluster hierarchies (bread similarity groupings) remained consistent in the dendrograms created using the parameter categories. However, the similarity values varied, as expected (Table 3). It should be noted that these partial hierarchical analyses cannot replace the final dendrogram based on the entire set of descriptors, but they do show whether one group of descriptors has a greater influence on similarity than the others.

## 4. Discussion

### 4.1. Biocultural Diversity

For the first time, hierarchical cluster analysis has been applied to a categorization of flatbreads. This explorative analysis is a multivariate statistical approach to identifying natural groupings within complex datasets based on overall similarity. A key output is the dendrogram, which is a tree-like, visual representation of relationships among observations and the relative similarity at which clusters form [84]. Originally developed and applied in the field of anthropological sciences, this approach was adopted in biological sciences to analyze genetic, ecological, and phenotypic data [84]. The approach offers an innovative perspective in the field of food research and has been used only once previously to compare food samples in a study on types of filled pasta [85].

The greater the number of characteristics considered, the more reliable the statistical clusters. In the filled pasta study [85], 47 entries were applied to differentiate 28 samples. In the present study, 69 entries were used to compare four types of bread. The resultant dendrogram showed a Cophenetic Correlation Coefficient (CCC) value of 0.97 and a *p*-value of 0.001, confirming a statistically significant and robust result. The CCC is a metric of clustering quality which quantifies the degree of agreement between the original dissimilarity matrix, based on the Jaccard index, and the cophenetic matrix derived from the clustering process. A reference threshold of 0.7 is commonly adopted as a criterion of adequacy, with lower CCC values indicating that the clustering method does not adequately summarize the structure of the dataset, and higher values indicating high correlation and, consequently, a highly accurate representation of clustering [86,87].

According to the dendrogram, each bread type had a distinct identity. To quantify, Somali *laxoox*/*canjeero* and Southern Arabian *lahoh* had a 64% similarity (that is, 36% diversity) to each other; each was 53% similar (47% diverse) to Ethiopian *injera*; while the Somali, Southern Arabian, and Ethiopian breads were 41% similar (59% diverse) to Sudanese *kisra* (Figure 8). These unique identities lay the foundations for policies to leverage their nutritional value for public health, to leverage investment in drought-resistant grains in the Horn of Africa and Southern Arabia, to ensure the continued, sustainable use of indigenous African and Arabian grain varieties, and to protect the qualities and unique identities of these breads, which tend to erode over time with globalization.

The physical–nutritional and process-related parameters contributed to similarity more than the ingredients (Table 3). Parallel similarities were observed in the production stages between Somali *laxoox*/*canjeero* and Southern Arabian *lahoh.* These findings are consistent with a previous investigation showing a close relationship between Somali *laxoox* and Sana’ani style Yemeni *lahoh*, assessed through principal component analysis of physical–chemical and nutritional data [27]. Furthermore, several commonalities appeared among all the studied breads apart from *kisra*. These included texture, appearance (e.g., shape, the presence of “eyes”), the use of pre-gelatinized dough, and the singular purpose of the batter (e.g., for breadmaking only, as opposed to *kisra*’s multi-purpose batter for bread, porridge, and potentially the drink *hulu-mur*).

Comparisons of breads from the Horn of Africa and Southern Arabia are few, and none have thoroughly analysed the four bread types. Lyons and D’Andrea [21,34] drew comparisons between *taita* (*injera*) and Yemeni *lahoh* and their ingredients, tools, and preparation methods, highlighting similarities in the habit of boiling part of the batter until the starch gelatinizes, helping “eye” formation. A common origin of *kisra* and *taita* (*injera*) was instead suggested by Dirar [26], based on similarities between the circular ceramic *doka* used to prepare *kisra* and *mogogo* (Tigray) or *mitad* (Amharic) griddles. However, the former was largely replaced by metal rectangular pans (*saj*) by the 19th century, so the necessary evidence of a shared history between *kisra* and *taita* (*injera*) may be lost. Matthews [22], through an in-depth examination of early griddle plates from three regions—Central Sudan, Eastern Sudan, and the Tigray highlands of Ethiopia and Eritrea—remarked upon similarities in the griddle-based techniques used to bake *kissra* and *injera*, arguing that regional differences are not stable and that recent variations may obscure more ancient connections. With the aim of presenting a holistic, modern-day comparative perspective, this study considers recent evidence and information from the previous one to two generations, within the 20th century. Similarities and differences among Somali *laxoox*/*canjeero*, Ethiopian *injera*, Sudanese *kisra*, and Yemeni/Saudi *lahoh* are discussed in greater detail in the following sections.

### 4.2. Technical Features of the Breads

#### 4.2.1. Bread Appearance and Quality Characteristics

Several visual quality characteristics are similar across the breads, including softness and pliability. All are circular or oblong shaped except for local variations (e.g., *lahoh Ariiqy*). *Kisra* is unique for its absence of “eyes” and lack of spongy texture. A cursory analysis of thickness versus diameter ranges shows a loosely inverse relationship (Table 1); the smaller Somali and Southern Arabian breads are on average thicker while the larger Ethiopian and Sudanese breads are on average thinner. The difference between the thickest recorded *injera* (6 mm) and the thinnest recorded *kisra* (1 mm) is notable.

Colours range from medium brown (due to use of red sorghum) to yellow/tan to white depending on the bread formulation. *Injera*/*taita* has a distinctly reddish hue when made with red teff. Colour is consistent throughout each piece of bread, although Yemeni *lahoh* may be left long on the griddle to brown the underside and a local variation featured high-contrast stripes. Somali *laxoox*/*canjeero* features a distinct spiral that is absent or inconsistent in other breads.

#### 4.2.2. Ingredients

The geographical areas of research feature similar but distinct cultivation patterns that favour ancient, dual purpose (human and animal consumption), dryland crops such as sorghum and millets, although other crops, such as teff, are cultivated and may feature in the flatbreads. Likewise, the study areas show increased use of wheat and alternative grains over the last half-century, in some cases to achieve priority quality characteristics (e.g., texture or colour) with less labour (e.g., to negate the need for pre-gelatinized dough) or at a lower cost (e.g., the use of rice in *injera* to render it light in colour).

**Figure 14:**
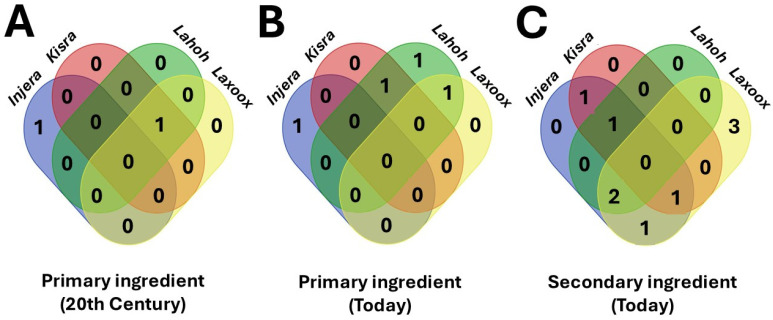
Venn diagrams showing the number of ingredients shared by four flatbreads (Sudanese *kisra*, Southern Arabian *lahoh*, Ethiopian *injera*/*taita*, and Somali *laxoox/canjeero*) over time. The number of primary ingredients used in the 20th century (**A**), today (**B**) and secondary ingredients (**C**) are shown. The overlapping areas indicate the number of ingredients common to multiple sets, while the individual areas contain ingredients exclusive to a single set. Keys: violet = *injera*; red = *kisra*; green = *lahoh*; yellow = *laxoox*.

The analysis of grains used in these flatbreads reveals commonalities among them that are not emphasized in studies which focus on customary prevalence or popular perception. Venn diagrams (Figure 14A–C) help in visualizing the number of ingredients shared among the examined bread types, integrating data reported in Table 1. Separate diagrams show the number of primary ingredients (frequency of use) used in the 20th century and today, as well as the secondary ingredients.

In the 20th century, only two types of cereals were used as primary ingredients: sorghum and teff. The former was used in three types of bread (*laxoox*/*canjeero*, *lahoh*, and *kisra*), while teff may only have been used in *injera* (Figure 14A, Table 1). Today, there are more types of cereals used as main ingredients, such as millet and wheat. The primary ingredient common to *lahoh* and *laxoox*/*canjeero*, wheat, has replaced sorghum and was also found in modern-day *kisra* (Figure 14B, Table 1). Since wheat is mainly imported in the areas of study, this change is a sign of globalization. No ingredient was common to all four types of bread considered, either in the past or today.

A total of nine secondary ingredients were recorded (Figure 14C). These were mainly cereals (the same ones that could be primary ingredients in other types of bread), but also legumes. Five of these were common to at least two types of bread. Two secondary ingredients were common to *laxoox*/*canjeero*, *lahoh*, and *injera* (corn and barley).

Originally, all these breads were likely made with gluten-free flours from locally available cereals (e.g., sorghum, teff). The absence of gluten made griddle preparation necessary as the batter lacked the viscoelasticity of a dough suitable for oven baking [17,34]. Sorghum, millet and wheat are used today in all the studied breads to greater and lesser degrees, including in *injera* and *kisra*, which are conventionally still associated with specific grains. Sorghum is a key ingredient in Somali, Sudanese, and Southern Arabian flatbreads and is also found in *injera*. As such, it represents agro-culinary ties between Africa, where it was domesticated, and Southern Arabia. Teff, commonly associated with *injera*, is likewise present in Somali *laxoox* in Ethiopia’s Somali State [28]. In terms of homogeneity within breads, *injera* and *kisra* are usually prepared with a single cereal. Whereas some breads may include a blend of varieties from a single cereal, *kisra* preparation typically involves the use of either red or white sorghum per batch, with a lower preference for red pericarp cultivars [88]. All the studied breads may include additional ingredients for reasons such as taste, texture, colour, or perceived health benefits. Less common ingredients include rice flour, cowpea, and groundnut.

However, it should be noted that, since millet constitutes a large group of species and a similar situation occurs for barley (two-row, six-row, hulled, and naked) and wheat (soft and hard), further studies would be needed to identify the exact botanical species used in these breads, both today and in the past, combining botanical data, empirical observations, specific field research, and written historical sources. The availability of additional information would allow for a better understanding of crop movements in the region [89] and could enable further refinement of the classification of bread types.

Room-temperature water is the sole liquid ingredient in the studied breads, although Somali cooks may use hot tea (black tea flavoured with various spices), typically prepared alongside *laxoox*/*canjeero* for breakfast, to speed fermentation before baking if the batter did not ferment overnight to their satisfaction. Salt is a typical ingredient in all breads except for *kisra*, while other additional flavourings (fenugreek, nigella seed, coriander, cumin, sugar, *za’atar*) are somewhat common in Somali *laxoox*/*canjeero* but were rarely reported in other breads.

Interestingly, the emblematic ingredients of these breads are evolving. Several studies have been undertaken during the last half-century on the partial or full use of alternative cereals and other ingredients in *injera*, *kisra* and *canjeero* for nutritional and/or quality enhancement, such as amaranth [65], baobab fruit pulp [64], carrot pulp [51], sweet potato [90], bonavist bean (*Dolichus lablab*) and white bean (*Phaseolus vulgaris*) [73], bambara groundnut (*Voandzeia subterranea*) [63], chick pea and soybean [91] and even oyster mushrooms and spirulina [92].

#### 4.2.3. Batter Preparation

A comparison of the essential production steps of each bread is presented in Figure 7. To prepare the breads, cooks combine one or more cereal flours with water. Somali *laxoox*/*canjeero* and Ethiopian *injera* have more liquid batters, with flour-to-water ratios of 1:2. *Kisra* and Southern Arabian *lahoh* have similarly liquid or more viscous batters, with reported ratios ranging from 1:2 to 2:3. In all cases, to speed fermentation, especially in colder climates and seasons, microbial starters may be added to the batters, either in the form of a portion of the previous batter or simply a coating of the previous batter remaining in the fermentation container. In the cases of Somali *laxoox*/*canjeero* and Southern Arabian *lahoh*, microbial starters are tools to optimize fermentation as opposed to essential components of the batters. Modern preparations of these breads may include instant yeast.

Some of the flatbreads are traditionally (previous one to two generations) based on non-glutenous cereals and therefore required pre-gelatinization to achieve a soft and chewy texture which is, today, a quality characteristic of these breads. In Ethiopia, *injera* is consistently prepared using *absit*; wheat flour was present in modern preparations of all breads except for *injera*. Pre-gelatinized dough in Somali *laxoox/canjeero* and Southern Arabian *lahoh* appears to be waning as the integration of wheat flours increases. However, the use of *cajiin* continues in South–Central Somalia and the use of *shorba* continues in some rural areas of Yemen where wheat flour may be more difficult to access or where traditional preparations are less influenced by globalization/urbanization. Evidence was not found of pre-gelatinized dough in Sudanese *kisra*; notably, the texture of baked *kisra* is unique among the studied breads (thinner and decidedly not spongy).

Among the breads which incorporate pre-gelatinized dough, the technique differs. *Cajiin* was used one to two generations previously in Somaliland and Somalia in urban areas where it was commercially available, and in rural areas of Somaliland where it was prepared manually. In both cases, the pre-gelatinized dough was prepared separately from the main slurry and then incorporated for fermentation [28]. In the traditional Southern Arabian *lahoh* preparation, the *shorba* and slurry are prepared and fermented separately and then combined before baking. In the case of *injera*, the *absit* is created using a portion of long-fermented batter thinned with water, heated and incorporated back into the batter for the second fermentation. All batters require thorough mixing before fermentation, either by hand (traditional and rural preparation for all breads and found today in Somaliland), whisk, or household blender.

*Kisra* is unique in representing one output of an efficient, multi-purpose fermented sorghum preparation. The dough is used directly after fermentation to produce *aseeda* or thinned with water into a pourable batter to bake *kisra*. Further, in what the authors call a traditional preparation, Mariod et al. [93] report soaking, germinating, drying, and milling sorghum grains for the preparation of Sudan’s fermented *hulu-mur* drink; in their experiment, the same germinated grains were used to prepare *kisra*. While the germination step was not reported by other *kisra* sources, it may once have been part of a single process of preparing multiple dishes from a single “batch” of sorghum. A sorghum porridge known as *aseeda* is prepared in Southern Arabia (Yemen and Saudi Arabia), although evidence of similar co-production was not found.

#### 4.2.4. Fermentation

Traditional fermented foods have played important roles in global diets for centuries, including in African [39,94,95,96,97,98] and Arab countries [18,99]. All the studied flatbreads rely on the use of a sour microbial starter, a complex ecosystem composed of various yeasts and lactic acid bacteria (LAB), which contribute to the formation of lactic acid, acetic acid, and several flavour-responsible volatile compounds, such as alcohols, aldehydes, ketones, and esters, as typical by-products of fermentation [49,67,100]. Fermentation provides nuance of flavour, an inexpensive means of preservation, and enhances the nutrition and digestibility of raw ingredients [101]. Yet, the sour flavour lent by fermentation is appreciated to different degrees in communities where fermented foods are regularly consumed. A preference for sour tastes depends on intensity and context [102]. A fondness for acidic foods may have predisposed our ancestors to control rotting, i.e., fermentation, for favourable outcomes [103]. Bikila et al. [36] argue that sour taste, according to the duration of fermentation, is an important distinguishing factor among *injera* flatbread subtypes, and their classification is formal (i.e., carrying names such as *aflegna* and *komtata injera*). Informal subtypes and variations among the other studied breads are found at different levels (e.g., household or regional). For example, in Somalia and Somaliland, the household cook adjusts fermentation time and sour flavour according to household preferences. In Sudan, where the sour flavour of *kisra* is “*particularly enjoyed*” [62], a cook may consider the sourness of accompanying dishes and balance that of *kisra*. In Southern Arabia, sour taste is similarly regional but also seemingly latitudinal along the Yemeni Tihama and Saudi Hijaz. Southern Taiz (Yemen) *lahoh* is known to be less sour than the Northern Al-Mahwit (Yemen) *lahoh*, while further north in Jizan (Saudi Arabia) the flatbread is extremely sour.

In addition to the positive effect on flavour, acidification induced by the starters reduces the amount of antinutrients, especially phytic acid, as observed in the Arabian *lahoh* [57]. Ashenafi [49] studied the appearance of an acidic yellow liquid on the surface of the batter during *injera* fermentation, which is mostly discarded while a small amount is kept for successive breadmaking. Discarding the liquid layer results in the loss of soluble compounds (amino acids, sugars and minerals) and especially in the loss of a large portion of microorganisms. Analyses showed that the yeasts most prevalent in the yellow fluid belong to the genera *Candida* and *Pichia*, so the remaining *Saccharomyces* and *Torulopsis* become the dominant microbiological flora of the batter during continued fermentation [49].

Apart from Ethiopian *injera*, unique among the studied breads for its two-stage fermentation, the breads traditionally ferment in one long stage. Today, the same breads may be prepared using modern ingredients (e.g., chemical or instant yeast) and techniques (e.g., a shorter fermentation) for the sake of efficiency and/or to reduce labour. Experimental trials to reduce *kisra* fermentation time to four hours by using *Lactobacillus* and *Saccharomyces* cultures to meet the needs of commercial production were carried out by Ali and Mustafa [40]. Modified fermentation, however, may impact bread technological quality and sensory properties. A recent study [36] found that fermentation heavily influences three essential quality characteristics of *injera*, both in combination with temperature (e.g., rollability) as well as independent of it (e.g., number of “eyes” and extensibility).

#### 4.2.5. Shaping and Baking

All breads except for *kisra* are shaped by pouring batter in a circular motion onto the griddle, spiralling from the centre outward or the opposite. Cooks in the mid–late-20th century Sana’a, per one expert, used small gourds with holes cut in the bottom to scoop and release batter onto the griddles; in a separate expert demonstration, a hole was punctured in the bottom of an empty, clean can of tuna fish and used in the same way. In most cases, only the underside of the bread is directly exposed to the griddle. *Kisra* and Southern Arabian *lahoh* remain uncovered during baking, whereas the other breads are typically covered by metal or woven lids, and cooked under the effect of steam.

All the studied breads are traditionally prepared using griddles rather than ovens, a technical choice correlated with the performance characteristics of the ingredients, which were originally gluten-free. Charcoal or wood stoves of various proportions are used, shaped to suit the griddle sizes, or gas stoves. Except for Sudanese *kisra*, the griddles are all circular. A study on *injera* baking technologies [29] found that the quality of *injera* baked on a metal pan is inferior to that baked on a clay *mitad*; the greater thermal conductivity of the former generates fewer “eyes” in the flatbread and causes more burning. Ethiopian *mogogo/mitad* are on average 60 cm in diameter [21] while ceramic griddles in Yemen can reach up to 75 cm [59]. The Somali *laxoox*/*canjeero dhaawe* and Saudi *lahoh al-dawa* are distinct among the selected flatbreads for their smaller size (16–20 cm). Interestingly, a second-to-fifth century CE ceramic griddle found at Aksum measured 19 cm in diameter [46], closer to the modern Somali griddle size; griddle diameter has increased since the Aksumite period and may reflect women’s efforts to feed larger families or reduce cooking times [104], among other theories [21]. Indeed, the relative size of griddle plates across Northeast Africa (Egypt; Northern, Western, Central, Southern, and Eastern Sudan; Northern Ethiopia) increased in size starting from the beginning of the first millennium BCE [22]. Southern Arabian *lahoh* has been adapted to accommodate smaller frying pan sizes suitable for conventional home gas or electric stoves per the many recipes and videos available online; a range of griddle sizes are used depending on household size, purpose (e.g., retail sale or home consumption), and local tradition.

### 4.3. Consumption Patterns and Social Function

All the breads are staples of the local diet and have very versatile consumption patterns. Somali *laxoox*/*canjeero* is part of the daily diet across present-day Somalia (where it is known as *canjeero*) and Somaliland (where it is known as *laxoox)* as well as in Somali areas of Djibouti, Ethiopia, and Kenya. Ethiopian *injera/taita* is a traditional food of the Amhara and Tigray peoples. In Yemen, *lahoh* appears at almost every meal, especially in rural areas [58,105]. Sudanese *kisra,* also found in South Sudan and Chad, is the most popular bread in Sudan [26,60,92], although less popular in the north (where *al-gorassa* flatbread is more common) and west (where *aseeda* porridge is preferred).

While each has its distinct trends, most of these breads are eaten on a regular basis and at potentially any meal. Somali *laxoox*/*canjeero* is a typical breakfast food but can be served at additional meals. Each of the studied breads are eaten with savoury liquid or semi-liquid dishes, such as stews and sauces, and used as utensils. The breads may be served underneath—as on a communal platter—or alongside the main dish(es). Sharing from a communal platter, often practiced, symbolizes unity, respect, and profound hospitality, with flatbread playing a role as a unifying element. All the breads may also be eaten with dry foods, such as dried meats or pan-fried meats and vegetables. Only Somali *laxoox*/*canjeero* is routinely sweetened, e.g., with sugar and oil, although interviewed experts on Southern Arabian *lahoh* shared that it is sometimes eaten with honey or a sweetened clotted cream. Depending on household preference, all the studied breads may be prepared and eaten daily or less frequently.

These flatbreads show important cultural and social functions. Sudanese *kisra* is eaten at weddings, funerals, and other gatherings of social significance in addition to regular consumption. According to one expert, *kisra* “must be available” because many traditional dishes are served with it. All the studied breads are used in religious rituals or celebrations, such as during Ramadan and in Christian celebrations among Ethiopia’s majority Orthodox demographic. Transcending its role as sustenance, bread is a profound vehicle of social identity and personal history, often rooted in formative childhood memories. A poignant illustration is the rhythmic, vigorous hand-mixing of *laxoox*/*canjeero* batter. Traditionally a nightly task performed by women, this sound has been elevated in Somali popular culture to a romanticized auditory marker of nightfall [28]. *Injera*’s significance is even woven into early-life rituals; for instance, in Bahir Dar, Ethiopia, some families roll newborn babies in *injera* during baptism ceremonies, believing this act secures a bright future and good fortune for the child [47]. *Injera* is considered “central to the Ethiopian consciousness” [106], and the “most definitive bread of highland Ethiopian cuisine” [34].

Bread is an essential food of the Southern Arabian Peninsula. In the context of displacement, it carries a great symbolic significance. For example, *lahoh* represents “home” for the Yemeni diaspora [27]. *Sorghum bicolor* was among the earliest crops cultivated in Yemen [107] where the origins of *lahoh* are uncertain, although the bread is assumed to have “ancient roots” [105]. Studying the literature from the 10th century CE, the scholar al-Hasan al-Hamdani recorded that *lahoh* was a sorghum bread cooked on a griddle and was especially popular in the tribal areas of al-Hashid, al-Sharf, and al-Ahnum (Yemen), where it was said to be their best food [53]. In the 18th-century *Taj al-arus*, al-Zabidi notes that *lahoh* was eaten with milk or broth and was popular in the Tihama [78]. Al-Zabidi also asserted that *lahoh* was mainly found in Yemen at the time and was not, as previously claimed by a local sheikh in Saudi Arabia, more common in the Saudi Hijaz, demonstrating its contemporary presence along the Saudi western coastline [78]. Today, in addition to proverbs, multiple varieties of *lahoh* are known, and “countless” recipes exist [69]. Local experts shared that *lahoh* is particularly important in rural villages where a smaller variety of breads are available compared with urban centres. Rural villages continue traditional *lahoh* preparation (used one to two generations before today) whereas urban household cooks have modified preparation techniques. *Lahoh*, served with traditional foods, “*reflects Yemeni culture and traditions, and its manufacture requires skill and experience…it is considered an important part of the popular heritage and food culture in Yemen*” [59]. Bread is likewise part of Saudi culinary heritage [61]. A diverse range of traditional flatbreads feature techniques passed down through generations, including *khubs ahmar* which incorporates dates; *mifa*, cooked on the walls of a wood-fired *tannur*-style oven; *fatoot*, cooked on a hot, convex *saj* griddle; and fermented flatbreads such as the wheat-based *muqana* [108]. Per multiple sources, *lahoh* is a specialty specific to Jizan Province, Saudi Arabia ([71]; expert consultations), at the border of Saudi and Yemen, although it can be found in supermarkets elsewhere in Saudi Arabia during certain times of year such as Ramadan.

## 5. Conclusions

This study features a comprehensive comparison of a selection of fermented flatbreads in the Horn of Africa and Southern Arabian Peninsula to gain insight into the ways that different ingredients, tools, and processing techniques are used to create similar, though distinct, products. The information collected, partly bibliographically and partly through local experts, presents a picture of 20th-century biocultural characteristics of these breads. Although the period considered is relatively short, it represents an important moment of transition from bread formulations of only low- or non-glutenous grains to bread formulations that include refined wheat, to different degrees.

The main research question was to what extent the studied breads are similar or diverse, and in what ways, in a region of such intense historical engagement. Qualitative assessments of the breads were complemented by a hierarchical cluster analysis, applied here for the first time to flatbreads, to quantify relative similarities and differences among the breads. The key findings of the study are:

- Each bread has a unique biocultural identity as captured in qualitative and quantitative evidence, rooted in centuries of cross-regional exchange. All the breads are dietary staples and have important cultural and social functions thereby reinforcing community structures and advancing food sovereignty through the sustainable utilization of local agricultural resources.
- Somali *laxoox*/*canjeero* and Saudi *lahoh* share more similarities compared to Ethiopian *injera*, while Sudanese *kisra* is the most distinct among the breads.
- Of the 69 variables analyzed, ingredient-related variables contributed least to similarities among the breads. While primary and secondary ingredients used today are more diverse than they were in the 20th century, some are featured in more than one bread type while others remain unique to a specific bread type.
- Nevertheless, a notable trend across all breads is the shift from indigenous, gluten-free grains toward (imported) wheat, a sign of globalization that risks eroding the distinct character of each bread over time and underscores the need to preserve crop biodiversity and adapt to climate change.
- There are more similarities in process-related and physical–nutritional variables among the breads. These include the batter preparation, fermentation, and baking processes, as well as sensory quality characteristics and nutritional composition.
- Women are central to breadmaking and are active participants across the bread value chain, from grain cultivation and processing to home preparation and cottage industry.

One limitation of this study is the relatively small number of breads analyzed, although this also enabled a thorough comparison. Other flatbreads in the region are visually similar to those studied here, such as the Sudanese *gorasa* or the Saudi *qatayef*. They were not included in this study because they are not fermented; however, a future study could consider a broader selection of breads based on criteria such as similar quality characteristics. Future research would also benefit from a broader examination of flatbreads from areas with historical ties to the region, such as the Indian fermented flatbread *dosa* whose varieties include some of the common grains included here. Nevertheless, this article serves as a “map” of similarities and differences in flatbread formulation, production, and consumption, to inspire additional research, including into the specific environmental, agricultural, social, economic, cultural, or other constraints and preferences which have led household cooks in similar circumstances to develop unique techniques and flatbread products.

Another limitation is that the breadth of the nutritional characterization was constrained by the available data. Since these data were compiled from previous studies and the existing literature, variations in analytical conditions may affect the direct comparability of the results. Future research should focus on a wider sample set for each bread type, utilizing standardized analytical protocols to ensure full data comparability and a more robust nutritional profile. Furthermore, consideration of the nutritional value and potential of these breads—especially those which incorporate indigenous African grains, climate change-adaptive grains, and other ingredients with nutritional impact in their context—may yield inspiration for further research on consumer trends and preferences as related to nutritional benefit and policy and have practical implications in food policy.

The study also contributes to food heritage preservation by: (i) synthesizing the cultural and technical attributes of each bread, with potential applications in culinary education and industrial quality control; (ii) elucidating intra-bread diversity to limit reductive generalizations and highlight regional-, cultural-, and even household-level nuance of preparation; (iii) by using comparative analyses to draw out lesser-studied bread characteristics, and demonstrate the diversification within the last century of grains used for making fermented flatbread.

Taken together, these findings make a strong case for targeted policy action: to protect the identity and quality of these breads through geographic or cultural designation, to support continued use of climate-resilient indigenous grains in the face of increasing drought and import dependency, and to document traditional production knowledge before it is displaced by modernization. The biocultural lens applied here—treating technical and cultural characteristics as inseparable—provides a model for future research into flatbreads and fermented foods more broadly, including promising comparisons with related breads such as Indian *dosa*, and further investigation into the deep historical foodway connections between the Horn of Africa and the Arabian Peninsula.

## Figures and Tables

**Table 1:** Key descriptors of fermented flatbreads.

	Sudanese *kisra*	Ethiopian *injera*/*taita*	Somali *laxoox*/*canjeero*	Southern Arabian *lahoh*
**Bread appearance**				
Shape	Oval	Circular	Circular	Circular
Diameter (usual range)	30–45 cm (length)	50–60 cm	15–20 cm	15–30 cm
Thickness (usual range)	1–1.5 mm	2–6 mm	4–6 mm	2–6 mm
Surface appearance	Plain	Presence of “eyes” (bubbles)	Presence of “eyes” and spiralized pattern	Presence of “eyes”
Surface colour	Creamy white to light brown	Light yellowish brown to rusty red	Light beige to light brown	Off-white or yellow (Saudi Arabia); from similarly light to a deeper brown (Yemen)
Colour uniformity	Same in the upper and underside	Same in the upper and underside	Different in the upper and underside	Different in the upper and underside
Texture	Soft but not spongy	Spongy	Spongy	Spongy
Quality features	Rollable and extensible	Rollable and extensible	Rollable and extensible	Rollable and extensible
Intraregional variation in appearance/quality	*Kisra rhaheefa*/*rahifa* (very thin), *kisra-kerr* (thicker), and *kissra-habashsiya* (“Ethiopian-style” *kisra*, more porous and spongier, with “eyes”)	Colour variation according to the use of teff or not and the type of teff (highlands using teff vs. lowlands not always using; white teff less common since more expensive compared to more common red/brown teff)	Colour variation according to ingredient use (more regular use of whole grains and legumes in Somaliland compared to their relative lack of use in Somalia where refined wheat and maize are more common)	*Lahoh* Ariiqy (named from the Aruuq village near Taiz, Yemen), larger and thinner than usual range and showing dark parallel stripes with a typical “zebra” pattern; *lahoh* from the northern regions and the Tihama of Yemen is thicker and moister
**Ingredients**				
Primary ingredient (20th century)	Sorghum	Teff	Sorghum	Sorghum
Primary ingredient (today)	Sorghum	Teff	Wheat	Sorghum, millet, wheat
Secondary ingredient (today)	Millet, wheat	Sorghum, millet, maize, barley, wheat, rice	Sorghum, maize, millet, barley, teff, cowpea, groundnut	Barley, maize
Taste enhancers	None	Salt	Salt, sugar	Salt
Flavourings	Any of fenugreek, black cumin, coriander	Fenugreek	Any of fenugreek, black cumin, coriander	Any of fenugreek, *za’atar*
**Production stages**				
Batter preparation	Thicker mixture, dual use	Batter, only for breadmaking	Batter, only for breadmaking	Batter, only for breadmaking
Fermentation	Single fermentation for 10–24 h at ambient temperature; use of microbial starter; moderately intense sour flavour	Double fermentation (30–72 h first; 30 min–2 h second—at ambient temperature); use of microbial starter; use of pre-gelatinized fermented dough; very intense sour flavour (with subtype—*aflegna*, *komtata*—variations)	Single fermentation for 9.5 h on average at ambient temperature; use of microbial starter; use of pre-gelatinized dough; moderately intense sour flavour (with regional variations)	Single fermentation for 10–12 h (Yemen) or 18–24 h (Saudi Arabia)—at ambient temperature; use of microbial starter; use of pre-gelatinized dough; moderately intense sour flavour (with regional variations)
Shaping	Pouring in parallel strips or in a circular motion	Pouring in a circular motion	Pouring in a circular motion	Pouring in a circular motion or with parallel strips or other ways
Baking	On a metal sheet or a circular griddle, without flipping the bread, without a lid	On a circular griddle (cast iron, ceramic, aluminum, nonstick), without flipping the bread, using a lid	On a circular griddle (cast iron, ceramic, aluminum, nonstick), without flipping the bread, using a lid	On a circular griddle (cast iron, ceramic, aluminum, nonstick), without flipping the bread, without a lid
**Consumption pattern and social function**				
Meal	At any meal	At any meal	At any meal, but most commonly at breakfast	At any meal
Ways to serve it	Under or alongside other food	Under or alongside other food	Alongside other food	Under or alongside other food
Pairing with other foods	With savoury dishes	With savoury dishes	With savoury and sweet dishes	With savoury and sweet dishes
Social function (in addition to being eaten on a regular basis)	Eaten on special occasions such as weddings and funerals and to break the fast at Ramadan	Eaten on special occasions such as weddings and funerals and at religious celebrations	Eaten to break the fast at Ramadan	Eaten to break the fast at Ramadan
**Production capacity and organization**				
Size of production	Small-scale artisanal production	Small-scale artisanal production	Small-scale artisanal production	Small-scale artisanal production
Division of tasks between the genders	Central role of women in breadmaking	Central role of women in breadmaking	Central role of women in breadmaking	Central role of women in breadmaking

**Table 2:** Nutritional composition of fermented flatbreads (g/100 g dry matter).

	Carbohydrate	Protein	Fat	Fibre	References
Sudanese *kisra*	75.04–83.48	10.48–13.8	1.99–5.69	2.48–2.68	[63,64]
Ethiopian *injera*/*taita*	76.35–84.24	11.27–14.51	1.08–3.46	3.73–11.25	[47,65]
Somali *laxoox*/*canjeero*	80.63–84.68	12.47–15.94	2.47–4.11	4.72–7.03	[27]
Southern Arabian *lahoh*	81.81–83.33	12.78–15.06	3.14–3.89	6.13–6.29	[27]

**Table 3:** Effect of different groups of technical parameters on the similarity value observed among the examined breads. Bread clusters are as in Figure 13.

	Groups of Descriptors and Similarity Values (Jaccard Index)		
Compared Breads	Physical and Nutritional Parameters	Process-Related Parameters	Ingredients
Somali compared with Southern Arabian flatbread	0.77	0.73	0.36
Ethiopian compared with Somali and Southern Arabian flatbread	0.63	0.52	0.24
Sudanese compared with Ethiopian, Somali and Southern Arabian flatbread	0.28	0.34	0.21

## Data Availability

The original contributions presented in this study are included in the article/Supplementary Materials. Further inquiries can be directed to the corresponding authors.

## Supplementary Materials

The following supporting information can be downloaded at: https://www.mdpi.com/article/10.3390/foods15081333/s1, Table S1: Key vocabulary related to the production process of the selected fermented flatbreads: Somali *laxoox*/*canjeero*, Ethiopian *injera*, Sudanese *kisra*, and Southern Arabian *lahoh*; Table S2: Binary matrix (0 = No; 1 = Yes) with 69 entries, based on key descriptors of fermented flatbreads.
