# Supplementary material for: Comparative Analysis of Fermented Flatbreads in the Horn of Africa and the Southern Arabian Peninsula: A Picture of Biocultural Diversity
Source: Foods. 2026 Apr 11;15(8):1333. doi: 10.3390/foods15081333 (PMC13114710; doi:10.3390/foods15081333)
Supplement: Supplementary file 1 [file foods-15-01333-s001.zip › foods-4181251-supplementary.pdf]

Supplementary Tables

Table S1. Key vocabulary related to the production process of the selected fermented flatbreads: Somali *laxoox/canjeero,* Ethiopian *injera,* Sudanese *kisra,* and Southern Arabian *lahoh.*

	**Sudanese *kisra***	**Ethiopian *injera/taita***	**Somali *laxoox/canjeero***	**Southern Arabian *lahoh***
**Quernstone (saddle or rotary)**		Mouthan (19th c.)	Mixdin (*saddle*) and cali (*grinding baton*)	Madhana (*Yemen*)
**Batter mixing container**	Jirdaal (*plastic*), kantuush al ajiin (*wooden, traditional*), garae (*squash, traditional*)			
**Batter mixing instrument**	*Mufraka*			
**Fermentation container**	*Khomara*	*Bohaka*	*Caga qoshka*	
**Mixture of multiple grain/cereal/legume species**			*Budo*	
**Batter**	*Ajiin*		*Qosh*	المغرفه<br>*Ajeen*
**Microbial starter**	*Khmer*	*Irsho / Ersho*	*Dhanaanis*	*Dhanaanis* (Yemen)<br><br>الشتايه<br>*Al shataya* (Saudi Arabia)
**Pre-gelatinized dough**	N/A	*Absit*	*Cajiin*	*Shorba*
**Griddle**	*Doka / saj*	*Mitad* (*injera*) / *mogogo* (*taita*)	*Dhaawe / Bir canjeero*	*Dawa / sulla* (Yemen)<br><br>الطاوه<br>Al dawah (*Saudi Arabia*)
**Cloth to spread oil or ghee across griddle surface**			*Masaxaad*	*Masax* (Yemen)
**Tool to pour batter onto griddle**		*Jog* (Amharic); *Bourma* (Tigrinya)		
**Tool to spread batter across griddle**	*Ghegeriba/ gergeriba*			
**Griddle lid**		*Kidan / akimbalo / mog't*	*Halqaban / Doboolka bir canjeero*	

**Bread storage container**	*Raika* (made of date palm leaves)	*Mesob* (cylindrical, flat-bottomed, made of grass)

Table S2. Binary matrix (0 = No; 1 = Yes) with 69 entries, based on key descriptors of fermented flatbreads.

		**Sudanese** *kisra*	**Ethiopian** *injera/taita*	**Somali** *laxoox/canjeero*	**Southern Arabian** *lahoh*
**Bread appearance**	Diameter 15-20 cm	0	0	1	1
	Diameter 20-30 cm	0	0	0	1
	Diameter 30-45 cm	1	0	0	0
	Diameter 50-60 cm	0	1	0	0
	Thickness 1-1.5 mm	1	0	0	0
	Thickness 2-4 mm	0	1	0	1
	Thickness 4-6 mm	0	1	1	1
	Presence of "eyes" on the surface	0	1	1	1
	Presence of a spiralized pattern on the surface	0	0	1	0
	Circular shape	0	1	1	1
	Same colour in the upper and underside	1	1	0	0
**Bread texture**	Spongy	0	1	1	1
**Quality feature**	Rollability and extensibility	1	1	1	1
	Large intraregional variation of its appearance	1	1	1	1
**Primary ingredient (20th century)**	Sorghum	1	0	1	1
	Teff	0	1	0	0
	Millet	0	0	0	0
	Wheat	0	0	0	0
**Primary ingredient (today)**	Sorghum	1	0	0	1
	Teff	0	1	0	0
	Millet	0	0	0	1
	Wheat	0	0	1	1
**Secondary ingredient (today)**	Sorghum	0	1	1	0
	Teff	0	0	1	0
	Millet	1	1	1	0
	Wheat	1	1	0	0
	Maize	0	1	1	1
	Barley	0	1	1	1
	Rice	0	1	0	0
	Cowpea	0	0	1	0
	Groundnut	0	0	1	0
**Taste enhancers**	Salt	0	1	1	1
	Sugar	0	0	1	0
**Flavouring ingredients**	Fenugreek	1	1	1	1
	Black cumin	1	0	1	0
	Coriander	1	0	1	0
	Za'atar	0	0	0	1
**Nutritional composition**	Protein 10-15 g/100 g d.m.*	1	1	1	1
	Fat 1-6 g/100 g d.m.	1	1	1	1

	Fiber <3 g/100 g d.m.	1	0	0	0
	Fiber 3-8 g/100 g d.m.	0	1	1	1
	Fiber >8 g/100 g d.m.	0	1	0	0
**Batter density**	Fluid	0	1	1	1
**Purpose of batter use**	Only for breadmaking	0	1	1	1
**Fermentation**	Multistep (double) fermentation	0	1	0	0
	Fermentation longer than one day	0	1	0	0
	Ambient temperature	1	1	1	1
	Use of microbial starter	1	1	1	1
	Use of pregelatinized dough	0	0	1	1
	Use of pregelatinized fermented dough	0	1	0	0
	Strong sourness	0	1	0	1
	Moderate sourness	1	0	1	1
**Shaping and baking**	Pouring with a circular motion	0	1	1	1
	Pouring with parallel strips or other ways	1	0	0	1
	On a circular griddle (cast iron, ceramic, aluminum, nonstick)	1	1	1	1
	On a metal sheet	1	0	0	0
	Flipping (baking on two sides)	0	0	0	0
	Using a lid	0	1	1	0
**Consumption pattern**	At breakfast	1	1	1	1
	At lunch	1	1	1	1
	At dinner	1	1	1	1
	Preferably at breakfast	0	0	1	0
	Served under other food	1	1	0	1
	Served alongside other food	1	1	1	1
	Served with savory dishes	1	1	1	1
	Served with sweet dishes	0	0	1	1
**Social function**	In addition to being eaten on a regular basis, eaten on special occasions	1	1	1	1
**Production capacity and organization**	Small-scale artisanal production	1	1	1	1
	Central role of women in bread making	1	1	1	1

*d.m. = dry matter
